# Graphical modeling of binary data using the LASSO: a simulation study

**DOI:** 10.1186/1471-2288-12-16

**Published:** 2012-02-21

**Authors:** Ralf Strobl, Eva Grill, Ulrich Mansmann

**Affiliations:** 1 Institute for Medical Informatics, Biometrics and Epidemiology, Ludwig-Maximilians-Universität München, Munich, Germany; 2 Faculty of Health and Nursing Sciences, West Saxon University of Applied Sciences, Zwickau, Germany

## Abstract

**Background:**

Graphical models were identified as a promising new approach to modeling high-dimensional clinical data. They provided a probabilistic tool to display, analyze and visualize the net-like dependence structures by drawing a graph describing the conditional dependencies between the variables. Until now, the main focus of research was on building Gaussian graphical models for continuous multivariate data following a multivariate normal distribution. Satisfactory solutions for binary data were missing. We adapted the method of Meinshausen and Bühlmann to binary data and used the LASSO for logistic regression. Objective of this paper was to examine the performance of the Bolasso to the development of graphical models for high dimensional binary data. We hypothesized that the performance of Bolasso is superior to competing LASSO methods to identify graphical models.

**Methods:**

We analyzed the Bolasso to derive graphical models in comparison with other LASSO based method. Model performance was assessed in a simulation study with random data generated via symmetric local logistic regression models and Gibbs sampling. Main outcome variables were the Structural Hamming Distance and the Youden Index.

We applied the results of the simulation study to a real-life data with functioning data of patients having head and neck cancer.

**Results:**

Bootstrap aggregating as incorporated in the Bolasso algorithm greatly improved the performance in higher sample sizes. The number of bootstraps did have minimal impact on performance. Bolasso performed reasonable well with a cutpoint of 0.90 and a small penalty term. Optimal prediction for Bolasso leads to very conservative models in comparison with AIC, BIC or cross-validated optimal penalty terms.

**Conclusions:**

Bootstrap aggregating may improve variable selection if the underlying selection process is not too unstable due to small sample size and if one is mainly interested in reducing the false discovery rate. We propose using the Bolasso for graphical modeling in large sample sizes.

## Background

A common problem in contemporary biomedical research is the occurrence of a large number of variables that accompany relatively few observations. Thus, studying associations in high-dimensional data is not straightforward. Including all variables would result in a highly over parameterized model, computational complexity and unstable estimation of the associations [1]. This methodological problem has been solved for the domain of genomic medicine by using graphical modeling. Graphical models were identified as a promising new approach to modeling clinical data [2], and thereby the systems approach to health and disease.

A promising approach to describe such complex relationships is graphical modeling. Graphical models [3] provide a probabilistic tool to display, analyze and visualize the net-like dependence structures by drawing a graph describing the conditional dependencies between the variables. A graphical model consists of nodes representing the variables and edges representing conditional dependencies between the variables. In order to understand graphical models it is important to understand the concept of conditional independence. Two variables X and Y are considered conditional independent given Z, if f(x|y, z) = f(x|z). Thus, learning information about Y does not give any additional information about X, once we know Z.

Beyond association, this method has also been developed for estimating causal effects [4]. Recently, graphical modeling has been outlined as a tool for investigating complex phenotype data, specifically for the visualization of complex associations [5], dimension reduction, comparison of substructures and the estimation of causal effects from observational data [6]. Until now, the main focus of research was on building Gaussian graphical models for continuous multivariate data following a multivariate normal distribution [7]. A popular way to built Gaussian graphical models are covariance selection methods [8]. These methods are used to sort out conditionally independent variables. They aim to identify the non-zero elements in the inverse of the covariance matrix since the non-zero entries in the inverse covariance matrix correspond to conditional dependent variables. However, this method is not reliable for high-dimensional data, but can be improved by concentrating on low-order graphs [9].

Another approach to identifying the non-zero elements in the inverse covariance matrix has been proposed by Meinshausen and Buehlmann [10]. They propose the Least Absolute Shrinkage and Selection Operator (LASSO) [11] as a variable selection method to identify the neighborhood of each variable, thus the non-zero elements. A neighborhood is the set of predictor variables corresponding to non-zero coefficients in a prediction model by estimating the conditional independence separately for each variable. Meinshausen and Buehlmann showed that this method is superior to common covariance selection methods, in particular if the number of variables exceeds the number of observations. They also proved that the method asymptotically recovers the true graph.

The LASSO was originally proposed for linear regression models and has become a popular model selection and shrinkage estimation method. LASSO is based on a penalty on the sum of absolute values of the coefficients (*ℓ*_1 _-type penalty) and can be easily adapted to other settings, for example Cox regression [12], logistic regression [13-15] or multinomial logistic regression [16] by replacing the residual sum of squares by the corresponding negative log-likelihood function. Important progress has been made in recent years in developing computational efficient and consistent algorithms for the LASSO with good properties even in high-dimensional settings [17,18]. The so-called graphical lasso by Friedman et al. [19] uses a coordinate descent algorithm for the LASSO regression to estimate sparse graphs via the inverse covariance matrix. They describe the connection between the exact problem and the approximation suggested by Meinshausen and Bühlmann [10].

However, the determination of the right amount of penalization for these methods has remained a main problem for which no satisfactory solution exists [20].

The current methodology primarily provides solutions for continuous data. The relationship of binary data is difficult to identify using classical methods. Building binary graphical models for dichotomous data is based on the corresponding contingency tables and log-linear models [21]. The interaction terms are used to control for conditional dependencies. With a growing number of variables model selection becomes computationally demanding and quickly exceeds feasibility, thereby making the method difficult to adapt to high-dimensional data. For a fully saturated log-linear model one would need *2 p *parameters, with *p *being the number of variables. A common solution is to reduce the problem to first-order interaction where conditional independence is determined by first-order interaction terms.

The properties of the LASSO for logistic regression have recently been investigated. Van de Geer [22] focused on the prediction error of the estimator and not on variable selection. She proposed a truncation of the estimated coefficients to derive consistent variable selection. Bunea [23] showed the asymptotic consistency of variable selection under certain conditions for *ℓ*_1 _-type penalization schemes.

The adaptation of local penalized logistic regression to graphical modeling has been proposed by Wainwright [24]. Under certain conditions on the number of variables *n*, the number of nodes *p *and the maximum neighborhood size, the *ℓ*_1 _-penalized logistic regression for high-dimensional binary graphical model selection gives consistent neighborhood selection [24,25]. Wainwright et al. showed that a logarithmic growth in *n *relative *to p *is sufficient to achieve neighborhood consistency. Another new approach is based on an approximate sparse maximum likelihood (ASML) problem for estimating the parameters in a multivariate binary distribution. Based on this approximation a consistent neighborhood could be selected and a sensible penalty term can be identified [17].

However, when analyzing high-dimensional categorical data the main problem that there is no rationale for the choice of the amount of penalization controlled by the value of the penalty term for consistent variable selection still remains [20].

A possible solution might be to adapt bootstrap aggregating to these problems. Bootstrap aggregating (bagging) generates multiple versions of a classifier and aggregates the results to get a single enhanced classifier. By making bootstrap replicates of the original data multiple versions are formed, each acting as single learning data for a classification problem. Also, for linear regression it has been shown that bagging provides substantial gains in accuracy for variable selection and classification [26]. This idea has been carried further by Bach [27], resulting in the Bolasso (bootstrap-enhanced least absolute shrinkage operator) algorithm for variable selection in linear regression. Here, the LASSO is applied to several bootstrapped replications of a given sample. The intersection of each of these models leads to consistent model selection.

In this paper we adapted the method of Meinshausen and Bühlmann to binary data and used the LASSO for logistic regression to identify the conditional dependence structure. We applied bagging to improve variable selection, hence adapted the Bolasso. Performances are tested on a data set with known structure. This data set was simulated by Gibbs sampling [28]. We also applied graphical modeling methods to real-life data.

Objective of this paper was to examine the performance of the Bolasso to the development of graphical models for high dimensional binary data with various values for the penalty term and various numbers of bootstraps. We hypothesized that the performance of Bolasso is superior to competing LASSO based methods to identify graphical models. Specifically, the hypothesis was that the choice of the penalty is not critical as long as it is chosen sensibly, i.e. corresponding to a reasonable number of selected variables.

## Methods

### Data generation

This section presents an approach to simulate high-dimensional binary data from a given distribution and dimension by analyzing the results on a data set with known dependence structure. This analysis is performed in order to investigate the performance of the proposed methods.

All calculations are done using the statistical computing software R (V 2.9.0) [29].

We propose to generate the data via symmetric local logistic regression models and Gibbs sampling [28] as follows:

(1) Define the *p *× *p *matrix M of odds ratios as: *diag*(*M*) *= p*_*ii*_*, i = *1,*..., p *with *p*_*ii *_the baseline odds of variable $X^{\left(i\right)}m_{i}^{j}=p_{ij}$ with *P*_*ij *_as the corresponding odds ratio of *X*^(*i*) ^on *X*^(*j*) ^and vice versa.

(2) Start with k = 0.

(3) Choose starting values *x*^(*k*) ^= {*x*_1_,..., *x*_*p*_) according to *diag*(*M*).

(4) For each *i *in 1, ...., p generate new $x_{i}^{\left(k+1\right)}$ from a Bernoulli distribution $B\left(p_{i}^{*}\right)$ according to $\text{logit}\left(p_{i}^{*}\right)=\sum_{j\neq i}m_{i}^{j}\cdot x_{j}^{\left(k\right)}$

(5) Repeat for k = k + 1.After a burn-in phase the $x_{i}^{\left(k+1\right)}$ will reflect the true underlying binary distribution generating *X ****= ***(*X*^(1) ^, ..., *X*^(*p*)^) ∈ {0,1}^*p *^We chose a burn-in phase of 5000 iterations.

### Real-life data: Aspects of functioning in head and neck cancer patient

We evaluated the method to data measuring aspects of functioning of patients having head and neck cancer (HNC). The data originated from a cross-sectional study with a convenience sample of 145 patients with HNC. The data has previously been used for graphical modelling and has been published [30].

The patients had at least one cancer at one of the following locations: oral region, salivary glands, oropharynx, hypopharynx or larynx. Human functioning for each of the patients were assessed using the International Classification of Functioning, Disability and Health (ICF) as endorsed by the World Health Organization in 2001 [31]. The ICF provides a useful framework for classifying the components of health and consequences of a disease and can be used. According to the ICF the consequences of a disease may concern body functions (b) and structures (s), the performance of activities and participation (d) in life situations depending on environmental factors (e).

Thirty-four aspects of functioning were assessed for each of the patients 12 from the component Body Functions, three from the component Body Structure, 15 from the component Activity and Participation and another 4 categories from the component Environmental factors. For better interpretation of the graphs we show the 34 ICF categories together with a short explanation in Table 1.

**Table 1:** Short description of the ICF categories used for the graphical models on the HNC data

ICF Code	ICF Code description
b130	Energy and drive functions
b280	Sensation of pain
b310	Voice functions
b320	Articulation functions
b340	Alternative vocalization functions
b440	Respiration functions
b450	Additional respiratory functions
b460	Sensations associated with cardiovascular and respiratory functions
b510	Ingestion functions
b515	Digestive functions
b530	Weight maintenance function
b710	Mobility of joint functions
d175	Solving problems
d310	Communicating with - receiving - spoken messages
d315	Communicating with - receiving - nonverbal messages
d330	Speaking
d335	Producing nonverbal messages
d350	Conversation
d360	Using communication devices and techniques
d550	Eating
d560	Drinking
d570	Looking after one's health
d720	Complex interpersonal interaction
d760	Family relationship
d770	Intimate relationship
d850	Remunerative employment
d920	Recreation and leisure
s320	Structure of mouth
s430	Structure of respiratory system
s710	Structure of head and neck region
e125	Products and technology for communication
e225	Climate
e310	Immediate family
e580	Health services, systems and policies

### Principles of graphical models

**Figure 1:**
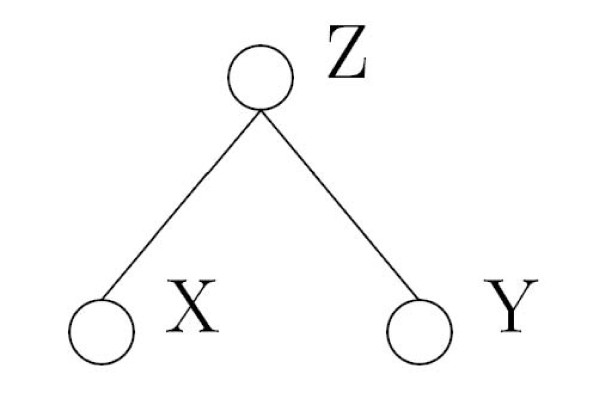
**An example for a simple graphical model**.

Consider *X ****= ***(*X*^(1) ^, ..., *X*^(*p*)^) ∈ {0,1}^*p *^as a p-dimensional vector of binary random variables. One way to represent the association structure between the elements of *X *in a random sample of i.i.d. replicates is an undirected binary graph. A graph *G*(*υ,ε*) consists of a finite set of nodes *υ*, representing the elements of *X*, and edges *ε *between these nodes. Each edge stands for an existing conditional dependence between two nodes. Hence, graphical modeling is based on the concept of conditional dependence and conditional independence. To understand graphical models it is fundamental to understand both of these concepts. Two events X and Y are independent, if *P*(*X *∩ *Y*) ***= ****P*(*X*) ⋅ *P*(*Y*) . Two events X and Y are conditional independent given Z if *P*(*X *∩ *Y *| *Z*) ***= ****P*(*X *| *Z*) ⋅ *P*(*Y *| *Z*) ⇔ *X *⊥ *Y*|*Z*. The relationship *X *⊥ *Y *| *Z *is represented in Figure 1.

A concept to describe a graphical model is via the neighborhood of each node. The neighborhood of node *X*^(*a*) ^*ne*_*a *_is defined as the smallest subset of *υ*, so that *X*^(*a*) ^is conditionally independent of all remaining variables, thus the neighborhood *ne*_*a *_is defined as:

(1) $$X^{\left(a\right)}⊥\left(\left\{X^{\left(i\right)};\forall X^{\left(i\right)}\in \upsilon /ne_{a}\right\}\right|ne_{a}$$

Two approaches define the edge set *ε*. First, an edge between x and y exists if and only if both nodes are an element in the opposite neighborhood, i.e. following the AND-rule:

(2) $$\varepsilon =\left\{\left(x,y\right)|x\in ne_{y}\wedge y\in ne_{x}\right\}$$

A less conservative, asymmetrical estimate of the edge set of a graph is given by the OR-rule:

(3) $$\varepsilon =\left\{\left(x,y\right)|x\in ne_{y}\vee y\in ne_{x}\right\}$$

A different definition of a neighborhood allows for a practical approach. For each node in *υ *consider optimal prediction of *X*^(*a*) ^given all remaining variables. Let *β*^*a *^∈ ℜ^(*p*-1) ^be the vector of coefficients for optimal prediction of *X*^(*a*)^. The set of non-zero coefficient is identical to the set of neighbors of *X*^(*a*) ^, thus:

(4) $$ne_{a}=\left\{b\in \upsilon :\beta_{b}^{a}\neq 0\right\}$$

We can regard this as a subset selection problem in a regression setting and to the detection of zero coefficients. The use of shrinkage as the subset selection tool used to identify the neighborhood and to estimate $\beta_{b}^{a}$ has become popular in recent years.

### Principles of LASSO for logistic regression

Given a set *of p *explanatory *X*^(1)^,..., *X*^(*p*) ^and a binary outcome *Y *the goal of logistic regression is to model the probability *π*_*i *_*= p*(*y*_*i *_*= *1| *x*_*i*_*) *by

(5) $$\text{log}\left(\frac{\pi_{i}}{1-\pi_{i}}\right)=x_{i}^{\prime }\beta \Leftrightarrow \pi_{i}=\frac{\text{exp}\left({x^{\prime }}_{i}\beta \right)}{1+\text{exp}\left({x^{\prime }}_{i}\beta \right)}$$

The maximum likelihood estimates of β can be found by setting the first derivative of log-likelihood function equal to zero, thus

(6) $$\frac{\partial l\left(\beta \right)}{\partial \beta }=s\left(\beta \right)=\overset{N}{\underset{i=1}{\sum }}x_{i}\left(y_{i}-\frac{\text{exp}\left(x_{i}^{\prime }\beta \right)}{1+\text{exp}\left(x_{i}^{\prime }\beta \right)}\right)=0$$

The LASSO is a penalized regression technique defined as a shrinkage estimator [11]. It adds a penalty term to equation (6), thus (6) is to be minimized subject to the sum of the absolute coefficients being less than a certain threshold value. Using the absolute values as condition yields shrinkage in some coefficients and simultaneously may set other coefficients to zero as has been shown by Tibshirani [11]. For each choice of the penalty parameter a stationary solution exists often visualized as a regularization path, i.e. the penalized coefficients over all penalty terms. LASSO reduces the variation in estimating β. Formally, the penalized logistic regression problem is to minimize:

(7) $$\overset{N}{\underset{i=1}{\sum }}x_{i}\left(y_{i}-\frac{\text{exp}\left(x_{i}^{\prime }\beta^{LASSO}\right)}{1+\text{exp}\left(x_{i}^{\prime }\beta^{LASSO}\right)}\right)+\lambda \underset{j}{\sum }\left|\beta_{\left(j\right)}^{LASSO}\right|$$

A mathematically equivalent expression of this problem is the formulation as a constrained regression problem, that is minimizing

(8) $$\overset{N}{\underset{i=1}{\sum }}x_{i}\left(y_{i}-\frac{\text{exp}\left(x_{i}^{\prime }\beta^{LASSO}\right)}{1+\text{exp}\left(x_{i}^{\prime }\beta^{LASSO}\right)}\right)$$

subject to $\underset{j}{\sum }\left|\beta_{\left(j\right)}^{LASSO}\right|<t$. In this paper, the variables are first standardized to zero mean and variance one. The penalty term *t *can be used to control the number of predictors, i.e. the size of the neighborhood.

### Binary graphical model using single LASSO regressions

The first method considered here to construct graphical models is based on an optimal penalty term to identify an optimal neighborhood. We consider three procedures for selecting optimal penalty, namely cross-validation (CV), Akaike Information Criteria (AIC) and Bayesian Information Criteria (BIC). These approaches have although been suggested in recent publications by Viallon [32] and Wang [33]. The first one is proposed by Goeman in the context of Cox regression [34], while the latter two are computational more efficient and well-known classical tools. BIC has shown to be superior as suggested by Yuan and Lin and demonstrated superior performance through simulation studies in Gaussian graphical modes [35,36]. Other possible approaches include the methods by Wainwright [24] and Banerjee [17] which control the rate of falsely detected edges in the graph. However, these penalties are too conservative as outlined in Ambroise et al. [37]. The LASSO and cross-validation are calculated using the efficient gradient-ascent algorithm as proposed by Goeman and are implemented in the 'penalized' package [38].

For all possible penalty terms, the performance of the resulting models is assessed either by cross-validation, AIC or BIC. The algorithm to identify a binary graphical model then proceeds as follows:

1. Estimate the coefficients ${\hat{\beta }}^{LASSO}$ in local penalized logistic regression models using each variable as outcome and the remainder as predictors for each *X*^(*i*) ^corresponding to an optimal penalty term *t.*

2. For each variable *a *define the neighborhood $ne_{a}^{*}$ as the set of variables *b *corresponding to non-zero penalized coefficients ${\hat{\beta }}_{b}^{a}:ne_{a}^{*}=\left\{b\in \upsilon :{\hat{\beta }}_{b}^{a}\neq 0\right\}$.

3. Define the set of conditional relationships (the edge set) E as: $E=\left\{\left(a,b\right)|a\in ne_{b}^{*}\vee b\in ne_{a}^{*}\right\}$.

### Binary graphical model using bolasso

Another method to construct binary graphical models is based on the Bolasso algorithm which takes advantage of bootstrap aggregating. Bootstrap aggregating, also called 'bagging', generates multiple versions of a predictor, e.g. a coefficient in a generalized linear model, or classifier. It constitutes a simple and general approach to improve an unstable estimator *θ(X) *with X being a given data set. Bagging is based on the concept of resampling the data {(*y*_*n *_, *x*_*n*_)*, n = 1,..., p*} by drawing new pairs $\left\{\left(y_{n}^{*},x_{n}^{*}\right)\right\}$ with replacement from the *p *original data pairs. For simple classification the bootstrap aggregating algorithm proceeds as follows:

(1) Generate a bootstrap sample b* with replacement from the original data. Repeat this process *B *times.

(2) For each data sample b* calculate the classifier θ*(b*).

(3) Count the times an object is classified $\mu_{x}=\underset{i=1..B}{\sum }\theta_{i}^{*}\left(b_{i}^{*}\right)$.

(4) Define the set of classified objects as *S *= {*y*: *μ*_*y *_≥ *π*_*cut*_} with 0 ≤ *π*_*cut *_≤ 1.

Using Bolasso as a basis, we can now construct graphical models. The algorithm proceeds as follows:

(1) Generate a bootstrap sample b* with replacement from the original data. Repeat this process B times.

(2) For each b*, estimate the coefficients ${\hat{\beta }}^{LASSO}$ in local penalized logistic regressions using each variable as outcome variable and the remainder as predictors for a penalty term *t.*

(3) For each variable *a *define the neighborhood $ne_{a}^{*}$ as the set of variables *b *corresponding to non-zero penalized coefficients ${\hat{\beta }}_{b}^{a}:ne_{a}^{*}=\left\{b\in \upsilon :{\hat{\beta }}_{b}^{a}\neq 0\right\}$.

(4) Calculate the percentage variable *b *is in the neighborhood of *a *in each bootstrap sample b* as $\mu_{b}^{a}$.

(5) Define the neighborhood of variable *a *as: $ne_{a}^{*}=\left\{b|\mu_{b}^{a}\geq \pi_{cut}\right\}$

(6) Define the set of conditional relationships (the edge set) E as: $E=\left\{\left(a,b\right)|a\in ne_{b}^{*}\vee b\in ne_{a}^{*}\right\}$.

Three parameters have to be chosen here:

• B: number of bootstraps

• t: penalty parameter

• *π*_*cut*_: cut-off value for the definition of neighborhood

In our study, we investigated the influence of these parameters for different sample sizes in a study based on simulated data as described earlier. We also considered using a cross-validated penalty as a basis for the Bolasso and refer to this approach by Bolasso-CV.

### Assessment of performance

We analyzed the performance of the methods by comparing the identified structure with a predefined known structure. Thus, each edge yielded by one method can be either correct or incorrect. A falsely identified (false positive) edge is an edge which is identified by one or both of the two methods but does not exist in the predefined structure. A falsely not identified (false negative) edge is an edge which is not identified by one or both of the two methods but does exist in the predefined structure. A correctly identified (true positive) edge is an edge which is identified by one or both of the two methods and which exists in the predefined structure. Likewise, a true negative edge is an edge correctly identified as missing.

We report the Structural Hamming Distance (SHD) and the Youden index (J). The SHD between two graphs is the number of edge insertions or deletions needed to transform one graph into the other. Thus, the number of changes needed to transform the graphical model identified by one or both of the two methods to the known structure defined by the matrix M. The SHD measures the performance of LASSO and Bolasso by counting the number of false positive and false negative edges.

It may occur that bagging causes the exclusion of all edges yielding a SHD equal to the number of true edges. This might reduce the error rate, but an empty model without edges is not always desirable, even if it has a low error rate. In order to assess both, the ability to find true positive and true negative edges, the Youden Index is more appropriate.

The Youden index is a function of the sensitivity (q) and the specificity (p) of a classifier and is defined as:

(9) J=q+p-1

Sensitivity is the proportion of true positive edges among all identified edges and specificity the proportion of true negative edges among all not identified edges.

Smaller values of the SHD indicate better choice, as do larger values of the J. Thus, a choice is to be preferred that yields small SHD at large J values.

**Figure 2:**
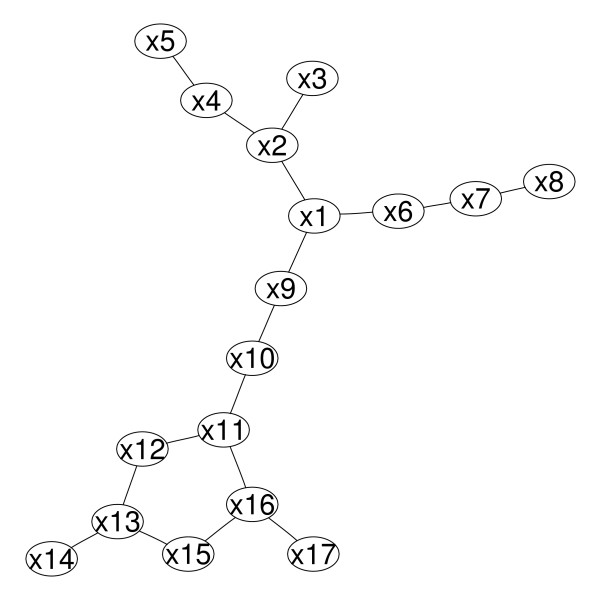
**The simulation setting for assessing the performance of the methods**.

We investigated the performances in a simulation setting which was motivated by a graphical model for real-life data [5] (see Figure 2). In this study functioning data for patients in the post-acute setting were analyzed using graphical modeling. The setting mimics a found subgraph in this graphical model. We additionally added two random variables having no interaction with the remainders to imitate a realistic scenario. The model in Figure 2 corresponds to a particular matrix of odds ratios, e.g. a smaller model with only 6 variables can be expressed by the matrix M:

$$M=\left(\begin{array}{cccccc} 1 & 2 & 2 & 1 & 1 & 1\\ 2 & 1 & 1 & 2 & 1 & 1\\ 2 & 1 & 1 & 2 & 1 & 1\\ 1 & 2 & 2 & 1 & 1 & 1\\ 1 & 1 & 1 & 1 & 1 & 1\\ 1 & 1 & 1 & 1 & 1 & 1 \end{array}\right)$$

We chose the penalty term to be either cross-validated, AIC or BIC optimal or to correspond to a certain neighborhood size ranging from one to the maximum neighborhood size, i.e. *l *∈ (1,2,...,18) in the original data. In addition, we varied the number of bootstrap replicates *B *∈ (40,80,120,160,200), the threshold for a variable to be included in a neighborhood *π*_*cut *_∈ (0.90,0.95,0.99,1.00) and the sample size *n *∈ (50,100,200,500,1000). Usually, *B *lies in the range of 50. However, the best choice is not clear, e.g. Bach investigated also *B ****= ***256, and may be important as the method itself is unstable. The choice of thresholds was also motivated by the work of Bach who proposed the soft Bolasso with a threshold of 0.90 as opposed to the Bolasso with a threshold of 1.00. We chose a wide range of values for B, thresholds and sample size to simultaneously study the performance of B and thresholds in small samples and in big samples. Ideally, for a sample size of 1000 the methods should perform with negligible error. In order to estimate model performances dependent on the parameters *π*_*cut*_*, B *and *l *and the interaction between *π*_*cut *_and *l *we calculated generalized linear models with either SHD or J as outcome variable.

## Result

### Simulations results

We calculated the Structural Hamming Distance and Youden Index for the simulation setting for each combination of *B, π*_*cut *_and *l*. We give the detailed results in table form in the electronic supplement (see additional file 1 and additional file 2: Summary statistics for the simulation study). Using generalized linear model yielded the following optimal regularization for minimal SHD: *π*_*cut *_***= ***0.90, *B ****= ***200 and *l ***= **5 . For a maximal J a larger neighborhood size is preferred: *π*_*cut *_***= ***0.90, *B ****= ***200 and *l ***= **10. It turned out, that the number of bootstrap replicates *B *has the least influence on model performance with higher *B *performing slightly better.

**Figure 3:**
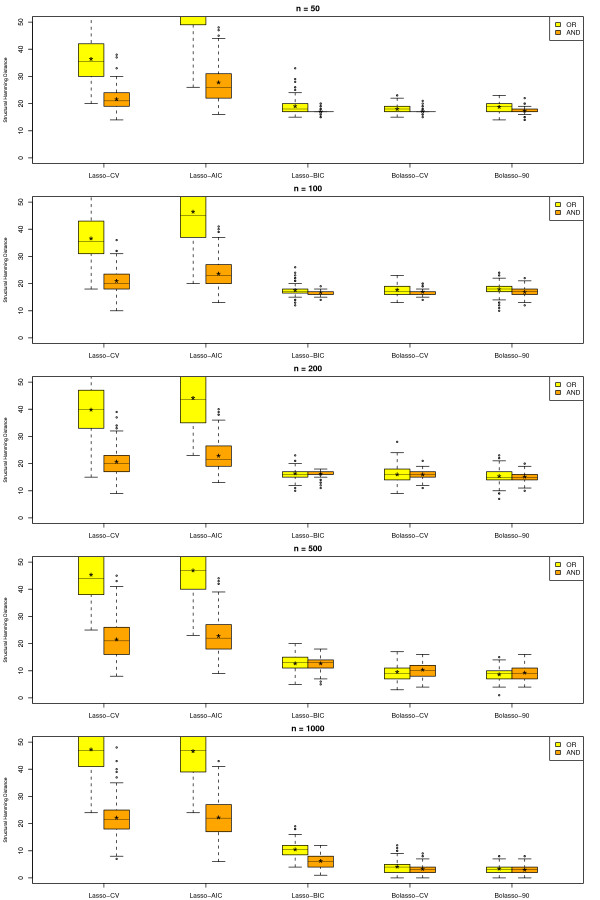
**Boxplot of Structural Hamming Distance (SHD) for all investigated approaches, different definitions of neighborhood (OR-rule and AND-rule) and sample sizes 50, 100, 200, 500 and 1000**. Yellow marks the OR-rule and orange the AND-rule. Lasso - CV represents cross-validated optimal penalty, Lasso - AIC represents AIC optimal penalty, Lasso - BIC represents BIC optimal penalty, Bolasso - CV represents Bolasso with cross-validated optimal penalty and Bolasso - 90 represents a Bolasso with a cut of 90% and neighborhood size 5.

**Figure 4:**
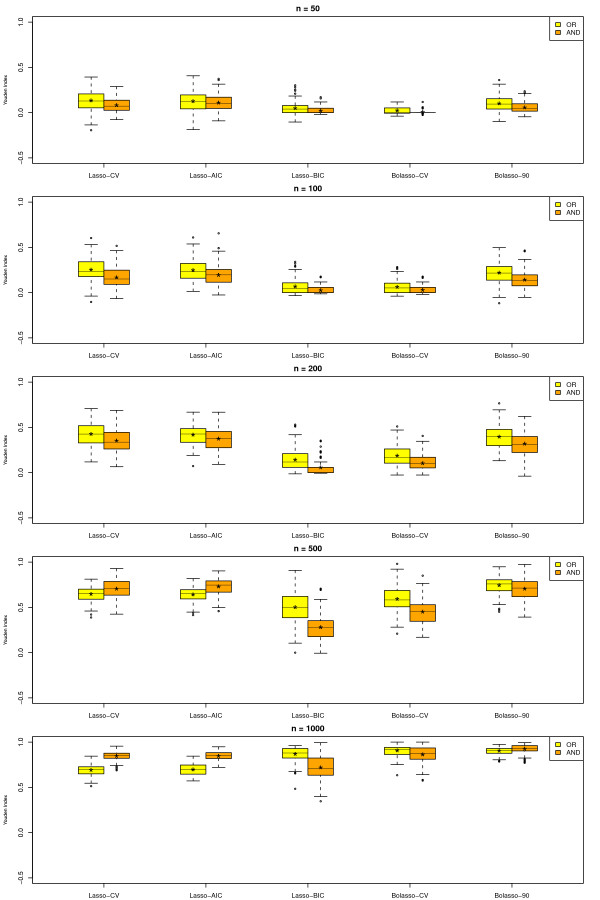
**Boxplot of Youden Index (J) for all investigated approaches, different definitions of neighborhood (OR-rule and AND-rule) and sample sizes 50, 100, 200, 500 and 1000**. Yellow marks the OR-rule and orange the AND-rule. Lasso - CV represents cross-validated optimal penalty, Lasso - AIC represents AIC optimal penalty, Lasso - BIC represents BIC optimal penalty, Bolasso - CV represents Bolasso with cross-validated optimal penalty and Bolasso - 90 represents a Bolasso with a cut of 90% and neighborhood size 10.

We give a summary of the results for SHD in Figure 3 and for J in Figure 4 for varying sample size. The figures show box plots for all methods, namely the Lasso-CV, the Lasso-AIC, the Lasso-BIC, the Bolasso-CV and the Bolasso with optimal neighborhood size and *π*_*cut *_***= ***0.90, the Bolasso-90. For each method we applied two different neighborhood definitions corresponding to the AND-rule and to the OR-rule. In the box plots * marks the mean performance.

Considering SHD as the main outcome renders Lasso-CV and Lasso-AIC as clearly inferior. For smaller sample sizes Lasso-BIC, Bolasso-CV and Bolasso-90 reject almost or all edges eading to a null model with SHD equal to 19. For sample sizes greater than 500 Bolasso-CV and Bolasso-90 are clearly superior to Lasso-BIC. Additionally, for each approach the AND-rule was superior to the OR-rule for most sample sizes.

A similar result can be seen when considering the Youden Index with the exception that Lasso-CV and Lasso-AIC are real contenders here, as they are not as conservative as the others, such gaining performance regarding sensitivity.

### HNC data

**Figure 5:**
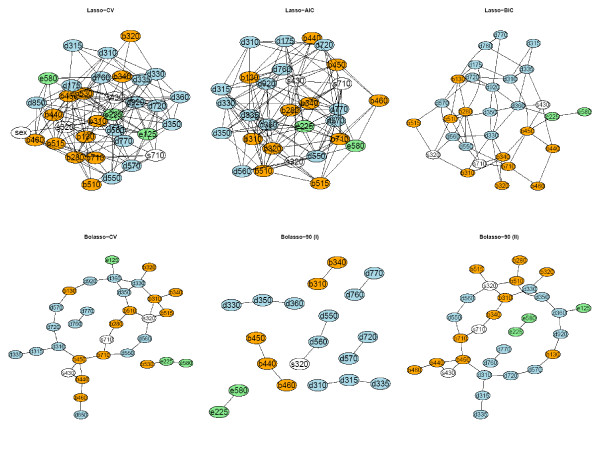
**Graphical models for the real-life data using the AND-rule**. ICF categories from the component Body function (orange), Body structure (white), Activities and participation (blue) and Environmental factors (green). Please, find the full descriptions of the ICF categories in Table 1.

**Figure 6:**
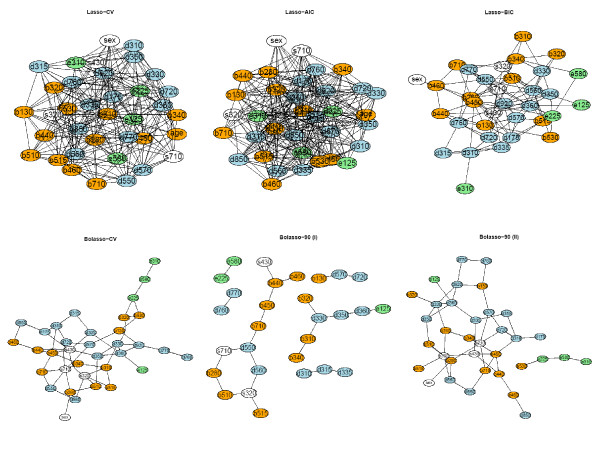
**Graphical models for the real-life data using the OR-rule**. ICF categories from the component Body function (orange), Body structure (white), Activities and participation (blue) and Environmental factors (green). Please, find the full descriptions of the ICF categories in Table 1.

Using these results we applied the method to the HNC data set using both the AND- and OR-rule. We give the results for the CV, the AIC and the BIC optimal penalty and for the Bolasso with a cut of 90%, 200 bootstraps and a neighborhood size of 5 (for optimal SHD), resp. 10 (for optimal J) in Figure 5 and 6. The color of the nodes correspond to the different ICF components: ICF categories from the component Body function are orange, Body structure white, Activities and participation blue and Environmental factors green. The full descriptions of the ICF categories are in Table 1.

In all models similar aspects can be seen. The CV and AIC optimal penalty term leads to a very complicated model, while the BIC criteria yielded reasonable results in terms of interpretability. The Bolasso-90 is the most conservative approach while using Bolasso-CV yielded similar results than the Lasso-BIC.

As a case in point, we describe the model for the Bolasso-90 with a neighborhood size of 10, i.e. the model with the highest performance regarding the Youden Index.

Similar to Becker et al. [30] we identified a circle-like association around the speaking capability, i.e. between d330 *Speaking*, b310 *Voice functions*, b320 *Articulation functions*, s320 *Structure of mouth*, b510 *Ingestion functions*, d350 *Conversation*, d360 *Using communication devices and techniques*. The latter had further associations to e125 *Products and technologies for communication *and d920 *Recreation and leisure*. The category s320 *Structure of mouth *had a meaningful connection to d560 *Drinking *which was further connected to d550 *Eating*. Furthermore, b510 *Ingestion functions *had an association to b280 *Sensation of pain*. On the left side of the graph we have a group around respiration functions, namely b440 *Respiration functions*, b450 *Additional respiratory functions*, b460 *Sensations associated with cardiovascular and respiratory functions *and s430 *Structure of respiratory system*. A further like path could be visualized between the categories d335 *Producing nonverbal messages*, d315 *Communicating with - receiving - nonverbal messages*, d310 *Communicating with - receiving - spoken messages*, d720 *Complex interpersonal interaction*, d570 *Looking after one's health *and b130 *Energy and drive functions*. The big circle is closed by the connection of b130 and d920 *Recreation and leisure.*

Many of these association structures were also present in the original work and are discussed in detail there [30].

## Discussion

We compared the performance of the Bolasso to the development of graphical models for high-dimensional data with known dependency structure. One of the main points of critique for graphical models is that the retrieved structures might not be statistically stable, since the results might depend on the choice of model parameters, and are susceptible to small changes in the data set [6]. Interestingly, we found that using a BIC penalty and Bolasso were both able to correctly identify predefined existing dependency structures.

We have analyzed several LASSO based methods to derive graphical models in the presence of binary data and compared their performance in detecting known dependency structures. All methods are taking advantage of penalized logistic regression models as a tool to identify the explicit neighborhoods. We could show that bootstrap aggregating can substantially improve the performance of model selection, especially in the case of large samples. Arguably, LASSO is inferior in certain situations because in a given LASSO coefficient path the optimal solution might not exist. A LASSO coefficient path is given by the coefficient value of each variable over all penalty terms. In contrast, bagging opens the window for a whole class of new models, because it selects all variables intersecting over the bootstrap samples. The intersection itself must not be a solution in any of the bootstrap samples.

In LASSO, the choice of the penalty often determines the performance of the model. Thus, the correct choice of the penalty term is important. However, in our study, the initial choice of penalization had surprisingly little impact on the performance of the model if bagging was applied and the penalty was chosen in sensibly. Similar results have been obtained with stability selection [20]. Stability selection is also based on bootstrap in combination with (high-dimensional) selection algorithms. It applies resampling to the whole LASSO coefficient path and calculates the probability for each variable to be selected when randomly resampling from the data.

In our study, bagging largely improved the performance of LASSO, but only by reducing the number of false positive and false negative edges. This, however, might lead to a conservative and underspecified model with a low number of edges, if any, especially in small samples.

Although the choice of the penalty term is not crucial when bagging is applied, a cut-off value for the definition of neighborhood has to be defined, and this arbitrary choice will determine the size of the graphical model. Reducing the cut-off value would include more variables at the expense of a higher false positive rate.

This study illustrates that in graphical modeling, it is essential not only to control the number of false positive and false negative edges but also the ability of a method to identify true positive edges.

## Conclusion

Bootstrap aggregating improves variable selection if the underlying selection process is not too unstable, e.g. due to small sample sizes. These properties have been shown on simulated data using various parameters. As a consequence, we propose using Bolasso for graphical modeling in large sample size cases as a real contender to the classical neighborhood estimation methods.

## Competing interests

The authors declare that they have no competing interests.

## Authors' contributions

RS computed the study; EG and UM contributed to the design of the study; all authors contributed to the analysis and writing. All authors read and approved the final manuscript

## Pre-publication history

The pre-publication history for this paper can be accessed here:

http://www.biomedcentral.com/1471-2288/12/16/prepub

## Supplementary Material

Additional file 1 **The table in the electronic supplement gives the exact numbers of the performances of each model**. The table gives summary statistics (mean, median, standard deviation, minimum and maximum) for the Structural Hamming Distance. Click here for file

Additional file 2 **The table in the electronic supplement gives the exact numbers of the performances of each model**. The table gives summary statistics (mean, median, standard deviation, minimum, and maximum) for the Youden Index. Click here for file
