# Supplementary material for: Graphical modeling of binary data using the LASSO: a simulation study
Source: BMC Med Res Methodol. 2012 Feb 21;12:16. doi: 10.1186/1471-2288-12-16 (PMC3305667; doi:10.1186/1471-2288-12-16)
Supplement: Additional file 2 — The table in the electronic supplement gives the exact numbers of the performances of each model. The table gives summary statistics (mean, median, standard deviation, minimum, and maximum) for the Youden Index. [file 1471-2288-12-16-S2.DOC]

|  | **Lasso-CV** | | **Lasso-AIC** | | **Lasso-BIC** | | **Bolasso-CV** | | **Bolasso-90** | |
| --- | --- | --- | --- | --- | --- | --- | --- | --- | --- | --- |
|  | **OR** | **AND** | **OR** | **AND** | **OR** | **AND** | **OR** | **AND** | **OR** | **AND** |
| **Sample Size = 50** | |  |  |  |  |  |  |  |  |  |
| Mean | 0,1336 | 0,0797 | 0,1241 | 0,1081 | 0,0481 | 0,019 | 0,0222 | 0,0065 | 0,1002 | 0,0562 |
| Median | 0,1285 | 0,0722 | 0,1255 | 0,1016 | 0,0393 | 0 | 0 | 0 | 0,0951 | 0,0458 |
| Min - Max | -0,5874 | -0,3659 | -0,5944 | -0,4637 | -0,4049 | -0,1895 | -0,1566 | -0,1436 | -0,4572 | -0,2808 |
| Std Dev | 0,1116 | 0,0784 | 0,1169 | 0,0903 | 0,064 | 0,0361 | 0,0413 | 0,0218 | 0,0891 | 0,0622 |
| **Sample Size = 100** | |  |  |  |  |  |  |  |  |  |
| Mean | 0,2518 | 0,1673 | 0,2457 | 0,1945 | 0,0666 | 0,025 | 0,0624 | 0,0293 | 0,2179 | 0,1406 |
| Median | 0,2368 | 0,1509 | 0,2311 | 0,1963 | 0,0458 | 0 | 0,0523 | 0 | 0,2162 | 0,1375 |
| Min - Max | -0,7056 | -0,5813 | 0,0141 - 0,6089 | -0,6799 | -0,3725 | -0,1895 | -0,3201 | -0,196 | -0,6138 | -0,516 |
| Std Dev | 0,123 | 0,1079 | 0,1176 | 0,1125 | 0,0748 | 0,0408 | 0,0662 | 0,0433 | 0,1155 | 0,0956 |
| **Sample Size = 200** | |  |  |  |  |  |  |  |  |  |
| Mean | 0,4262 | 0,3498 | 0,4185 | 0,3724 | 0,1421 | 0,056 | 0,1865 | 0,1036 | 0,3963 | 0,3192 |
| Median | 0,4257 | 0,3371 | 0,4267 | 0,3732 | 0,1176 | 0,0588 | 0,17 | 0,1047 | 0,3959 | 0,314 |
| Min - Max | 0,1188 - 0,7074 | 0,0657 - 0,6868 | 0,0726 - 0,6681 | 0,0917 - 0,6673 | -0,5428 | -0,3594 | -0,5359 | -0,4313 | 0,1314 - 0,7651 | -0,6601 |
| Std Dev | 0,1257 | 0,1287 | 0,1106 | 0,1294 | 0,1047 | 0,0663 | 0,1069 | 0,0813 | 0,1236 | 0,1244 |
| **Sample Size = 500** | |  |  |  |  |  |  |  |  |  |
| Mean | 0,6467 | 0,7057 | 0,64 | 0,7283 | 0,4992 | 0,2787 | 0,5913 | 0,4492 | 0,7419 | 0,7049 |
| Median | 0,652 | 0,7099 | 0,6494 | 0,746 | 0,5002 | 0,2779 | 0,5817 | 0,4544 | 0,7586 | 0,7128 |
| Min - Max | 0,3885 - 0,8117 | 0,4251 - 0,9286 | 0,4144 - 0,8182 | 0,458 - 0,9026 | 0 - 0,9087 | -0,7124 | 0,2093 - 0,9805 | 0,17 - 0,8499 | 0,4515 - 0,9481 | 0,3923 - 0,974 |
| Std Dev | 0,0815 | 0,1026 | 0,0804 | 0,0956 | 0,173 | 0,1435 | 0,1323 | 0,131 | 0,0922 | 0,107 |
| **Sample Size = 1000** | |  |  |  |  |  |  |  |  |  |
| Mean | 0,6911 | 0,8448 | 0,6958 | 0,8461 | 0,8669 | 0,7175 | 0,9061 | 0,863 | 0,9026 | 0,9241 |
| Median | 0,6948 | 0,8506 | 0,6948 | 0,8506 | 0,8795 | 0,7059 | 0,9217 | 0,8759 | 0,9091 | 0,9286 |
| Min - Max | 0,513 - 0,8442 | 0,6883 - 0,9545 | 0,5714 - 0,8442 | 0,7208 - 0,9481 | 0,484 - 0,961 | 0,3464 - 0,9935 | 0,6341 - 1 | 0,5752 - 1 | 0,785 - 0,974 | 0,7716 - 0,9935 |
| Std Dev | 0,0596 | 0,0496 | 0,0636 | 0,0491 | 0,0745 | 0,1378 | 0,0625 | 0,0862 | 0,0383 | 0,0476 |
